# Reproductive and Developmental Toxicity Assessment of Human Umbilical Cord Mesenchymal Stem Cells in Rats

**DOI:** 10.3389/fcell.2022.883996

**Published:** 2022-05-19

**Authors:** Xiaobo Li, Qijing Huang, Xiangxiang Zhang, Changfeng Xie, Muyun Liu, Yueming Yuan, Jianjia Feng, Haoyu Xing, Li Ru, Zheng Yuan, Zhiyong Xu, YaoXiang Yang, Yan Long, Chengfeng Xing, Jianping Song, Xiang Hu, Qin Xu

**Affiliations:** ^1^ Artemisinin Research Center, Guangzhou University of Chinese Medicine, Guangzhou, China; ^2^ Country Sci-Tech Industrial Park, Guangzhou University of Chinese Medicine, Guangzhou, China; ^3^ Shenzhen Beike Biotechnology Co., Ltd., Shenzhen, China; ^4^ Guangzhou First People’s Hospital, School of Medicine, South China University of Technology, Guangzhou, China; ^5^ Guangzhou Hospital of Integrated Traditional Chinese and Western Medicine, Guangzhou, China; ^6^ The First Affiliated Hospital of Guangzhou University of Chinese Medicine, Guangzhou, China

**Keywords:** mesenchymal stem cells, umbilical cord, reproductive toxicity, developmental toxicity, rats

## Abstract

**Objective:** Human umbilical cord mesenchymal stem cells (hUC-MSCs) have shown very attractive potential in clinical applications for the treatment of various diseases. However, the data about the reproductive and developmental toxicity of hUC-MSCs remains insufficient. Thus, we assessed the potential effects of intravenous injection of hUC-MSCs on reproduction and development in Sprague-Dawley rats.

**Methods:** In the fertility and early embryonic development study, hUC-MSCs were administered at dose levels of 0, 6.0 × 10^6^, 8.5 × 10^6^, and 1.2 × 10^7^/kg to male and female rats during the pre-mating, mating and gestation period. In the embryo-fetal development study, the pregnant female rats received 0, 6.0 × 10^6^, 1.2 × 10^7^, and 2.4 × 10^7^/kg of hUC-MSCs from gestation days (GD) 6–15. Assessments made included mortality, clinical observations, body weight, food consumption, fertility parameters of male and female, litter, and fetus parameters, etc.

**Results:** No hUC-MSCs-related toxicity was observed on the fertility of male and female rats, and no teratogenic effect on fetuses. hUC-MSCs at 1.2 × 10^7^/kg caused a mildly decrease in body weight gain of male rats, transient listlessness, tachypnea, and hematuria symptoms in pregnant female rats. Death was observed in part of the pregnant females at a dose of 2.4 × 10^7^/kg, which could be due to pulmonary embolism.

**Conclusion:** Based on the results of the studies, the no-observed-adverse-effect levels (NOAELs) are 8.5 × 10^6^/kg for fertility and early embryonic development, 1.2 × 10^7^/kg for maternal toxicity and 2.4 × 10^7^/kg for embryo-fetal development in rats intravenous injected with hUC-MSCs, which are equivalent to 8.5-fold, 12-fold, and 24-fold respectively of its clinical dosage in humans. These findings may provide a rational basis for human health risk assessment of hUC-MSCs.

## Introduction

Stem cells are known as “universal cells” because of their potential to proliferate, self-renew, and differentiate into various functional cells under certain conditions. In recent years, stem cell therapy become one of attractive therapeutic platforms for treatment of various human diseases and a hot area of current international medical cell therapy research and application (Gao et al., 2018; Goradel et al., 2018; Grochowski et al., 2018). Mesenchymal stem cells (MSCs) are a type of adult stem cells that originate from the early mesoderm, generally derived from placenta, fat, bone marrow, umbilical cord, and fetal internal organs, etc. MSCs, as a promising candidate for cell therapy of different diseases in humans and in animals, have been proved to be effective and safe by a large number of animal and clinical trials to treat of cardiovascular repair, lung fibrosis, spinal cord injury, bone and cartilage replacement and other diseases (Simones et al., 2018; Uder et al., 2018).

Umbilical cord is a rich source of MSCs for cell therapy. Unlike bone marrow stem cells, which require invasive and painful operation to harvest, a procedure that may cause hemorrhage, infection, and chronic pain in some cases. Human umbilical cord mesenchymal stem cells (hUC-MSCs) have many attractive advantages, including non-invasive collection procedure, low risk of infection, non-tumorigenesis, multi-potency, and low immunogenicity (Ding et al., 2015; Nagamura-Inoue and Mukai, 2016). At present, the MSCs drugs on the global market are basically derived from bone marrow and fat. Only South Korea has launched a hUC-MSCs product (Cartistem) for the treatment of degenerative arthritis and knee cartilage damage in 2012. To date, more than 50 clinical trials of hUC-MSCs treatment have been completed through retrieving the clinical trial registration website (ClinicalTrials.gov). The published animal and clinical trial results demonstrated that hUC-MSCs have several functions, including differentiation into terminal cells, immune regulation, paracrine effects, anti-inflammatory effects, anti-fibrotic effects, and regulating non-coding RNA, so it has good therapeutic effects in treating a variety of diseases clinically, such as diabetes and its complications, liver disease, systemic lupus erythematosus, arthritis, brain injury and cerebrovascular diseases, heart diseases, spinal cord injury, respiratory diseases, viral infections, etc. (Can et al., 2017; Colicchia et al., 2019; Gu et al., 2020; Reyhani et al., 2020; Xie et al., 2020). It is also notable that intravenous infusion of hUC-MSCs has shown promising results in coronavirus disease-2019 (COVID-19) clinical treatment for its comprehensive powerful immunomodulatory function. hUC-MSCs therapy can be considered a salvage and priority treatment option for severe COVID-19 (Colicchia et al., 2019; Feng et al., 2020; Meng et al., 2020; Shu et al., 2020; Zhang et al., 2020).

The safety of hUC-MSCs have been preliminarily verified in scientific research, preclinical, and clinical trials. Previous toxicity studies in animals have shown that hUC-MSCs have no obvious hemolytic reaction, immunotoxicity, sensitization, and tumorigenicity (Tian et al., 2019; Le et al., 2020). A single intravenous injection of hUC-MSCs at 1.0 × 10^8^/kg in mice did not cause acute toxicity (He et al., 2016; Wang et al., 2019). No treatment-related effects were observed in cynomolgus monkeys which were dosed intravenously with 1.0 × 10^7^/kg of hUC-MSCs for 98 days, 6 weeks or 6 months (Wang et al., 2012; Hai et al., 2016; He et al., 2017). However, in reproductive and developmental research of hUC-MSCs, more attention is paid to its therapeutic effects and mechanisms, such as recovering ovarian function and improving fertility in premature ovarian insufficiency/failure or natural aging mice/rats (Li et al., 2017; Lu et al., 2019; Ding et al., 2020; Yang et al., 2020; Zhao et al., 2020; Deng et al., 2021; Zhang et al., 2021), repairing the damaged endometrium in endometrial injury animal models (Zhang et al., 2018; Wang et al., 2021), reducing inflammatory response, and oxidative stress in testicular ischemia/reperfusion rats (Zhong et al., 2020), improving testicular failure in azoospermia mice (Abd Allah et al., 2017), information available on reproductive and developmental toxicity of hUC-MSCs in human and animals is scarce, making it difficult to assess the potential toxicity risk of hUC-MSCs. In the future, hUC-MSCs may be used in pregnant women and women with childbearing potential, its reproductive and developmental safety needs to be assessed. To address the lack of research in this aspect, the present studies were to investigate the potential adverse effects of hUC-MSCs on fertility and embryo-fetal development in rats and to provide a rational basis for human health risk assessment. hUC-MSCs have low immunogenicity and biological activity in rats (Ding et al., 2015; Zhu et al., 2015). Therefore, rats were selected for use as animal species in our studies.

## Materials and Methods

### Test Article and Preparation

The test article, hUC-MSCs (Lot No. 12802000011416000 and 12802000011402000) were manufactured and supplied by Shenzhen Beike Biotechnology Co., Ltd. (China). It was suspended in 5% DMSO culture medium solution and stored in liquid nitrogen. Stock solution of hUC-MSCs (passage 4 cells) was resuscitated in a water bath at 40°C, and centrifuged after being pipetted and washed with sodium chloride injection. The supernatant was discarded and washing was repeated twice. Finally, the cell precipitate was resuspended with cell preservation solution (5% human albumin compound electrolyte solution), and further diluted to obtain the dose formulations with required concentration according to the cell count results. All formulations were freshly prepared on the day of use and injected within 4 h. The test article preparation analysis for concentration verification, homogeneity, and stability was evaluated before the initiation of the studies.

### Animals and Maintenance

Specific pathogen free grade Sprague-Dawley (SD) rats were obtained from Hunan Silaikejingda Experimental Animal Co., Ltd. (China) with the laboratory animal production license number was SCXK (Hunan) 2016-0002. The animals were allowed to acclimatise for 5–7 days before the start of the study. Rats were maintained in an environmentally controlled room under standard laboratory conditions of room temperature (20–26°C) and relative humidity (40–70%) with a 12 h light/dark cycle. The certified commercial feed diet (Guangdong medical laboratory animal center, China) and drinking water were available ad libitum. All animal experiments were conducted in compliance with the Principles of Good Laboratory Practice (GLP) from National Medical Products Administration (NMPA), China, and were performed in the laboratory animal room of New South Center of Safety Evaluation for Drugs of Guangzhou University of Chinese Medicine, China [Chinese animal use license number: SYXK (Guangdong) 2018-0014]. Research protocols were approved by the Institutional Animal Care and Use Committee (IACUC) for animal care and use based on the 3R principle (Reduction, Replacement, and Refinement).

### Dose Selection Rationale

In the embryo-fetal development study, the dose levels of hUC-MSCs were selected on the basis of its proposed adult clinical dose (1.0 × 10^6^/kg) and the results from a previous internal study in monkeys. Repeated doses of hUC-MSCs at 1.0 × 10^7^/kg to cynomolgus monkeys may cause a decrease in red blood cell count, hemoglobin, and hematocrit, and an increase in total bilirubin (unpublished data). According to the equivalent body surface area conversion method, the above adult clinical dose and monkey dose were equivalent to 5.6 × 10^6^/kg and 2.47 × 10^7^/kg in rats, respectively. Therefore, 6.0 × 10^6^/kg and 2.4 × 10^7^/kg were selected as low and high doses. The middle dose was 1.2 × 10^7^/kg according to equal ratio principle.

In the fertility and early embryonic development study, dose design of hUC-MSCs was based on its proposed adult clinical dose (1.0 × 10^6^/kg) and the results from a pilot study in rats. After repeated intravenous administration of hUC-MSCs to rats at 7-days intervals, mortality was relatively high at 2.4 × 10^7^/kg and no death occurred at 6.0 × 10^6^/kg. And combined with the results of the embryo-fetal development study, part of the female rats died at high dose, so the high dose was adjusted down to 1.2 × 10^7^/kg. The low dose remain 6.0 × 10^6^/kg, which is close to the clinical equivalent dose. The middle dose was 8.5 × 10^6^/kg according to equal ratio principle.

### Experimental Design

In the fertility and early embryonic development study, male rats (approximately 5–6 weeks old and 200–264 g at randomization) and female rats (approximately 7–8 weeks old and 199–248 g at randomization) were randomly assigned to five groups of 25 rats per sex per group: saline control group, vehicle control group (cell preservation solution), low dose group (hUC-MSCs 6.0 × 10^6^/kg), medium dose group (hUC-MSCs 8.5 × 10^6^/kg) and high dose group (hUC-MSCs 1.2 × 10^7^/kg). All animals were administered by tail vein injection at a dose volume of 10 ml/kg, and the low, medium, and high concentrations of hUC-MSCs preparation were 0.6 × 10^6^/ml, 1.2 × 10^6^/ml, and 2.4 × 10^6^/ml, respectively. The period of administration for males was 9 weeks prior to cohabitation, for female rats was 2 weeks prior to cohabitation and continued until gestation day (GD) 6 for pregnant rats. Males were administered for 3 weeks, with an interval of 3 weeks, and continue to be administered for 3 weeks (once on D1, D8, D15, D43, D50, D57). Females were administered 3 times in 2 weeks (once on D50, D57, D64) and pregnant females were administered on GD6. Throughout the study, all animals were checked daily for clinical signs and abnormal behavior. Individual body weight was recorded twice per week, and food consumption was measured once per week. The pregnant females were weighed once every 3 days, and the food consumption was measured every 3 days. The vaginal smear of each female was made every day from administration (D50) to cohabitation (D60) to evaluate the oestrous cycle. The mating period was 14 days, and the mating and pregnancy rate of females were measured. After mating confirmation, males were sacrificed and necropsied, and the testes and epididymis were weighed, the sperm motility of epididymis, sperm count, and morphology were evaluated. Cesarean sections were conducted on GD15, and pregnancy status and numbers of corpora lutea, implantation sites, and placentae, and viable, dead and absorption fetuses were recorded for each pregnant female. At necropsy, ovaries and uterus were weighed and collected, and evaluated microscopically.

In the embryo-fetal development study, 50 male and 150 female rats of approximately 7–8 weeks old, weighing between 274 and 341 g (males) and 202–245 g (females) upon arrival. Males were used for mating, and 125 females were selected due to mating confirmation and the weight on GD0 was 205–296 g. The mated females were randomly divided into five groups of 25 rats per group: saline control group, vehicle control group (cell preservation solution), low dose group (hUC-MSCs 6.0 × 10^6^/kg), medium dose group (hUC-MSCs 1.2 × 10^7^/kg) and high dose group (hUC-MSCs 2.4 × 10^7^/kg). Animals were observed twice daily for clinical signs from the start of pregnancy (GD0) to the end of the experiment (GD20). Individual body weight and food consumption were recorded on GD0, 3, 9, 12, 15, 18, and 20. The pregnant females were administered by tail vein injection at a dose volume of 10 ml/kg, once on GD6, GD9, GD12, and GD15. The low, medium, and high concentrations of hUC-MSCs preparation were 0.6 × 10^6^/ml, 0.85 × 10^6^/ml, and 1.2 × 10^6^/ml, respectively. On GD20, the pregnant females were sacrificed and necropsied, numbers of corpora lutea, implantation sites, and viable, dead and absorption fetuses were counted. Viable fetuses were removed from the uteri and sexed, weighed, determinated length of body and tail, and examined for external, visceral and skeleton deformity.

For both studies, during the mating period, female rats were mated overnight with male breeder rats (1:1), and vaginal smear examination was performed the next morning for evidence of mating. The day on which spermatozoa were found in the vaginal smear was considered as gestation day 0 (GD0).

### Statistical Analysis

The data was analyzed by SPSS 19.0 statistical software and represented as mean ± SD, except where otherwise mentioned. If the variance was homogeneous, the measurement data (body weight and weight gain, food consumption, reproductive organ weight, and cesarean section data) were compared across treatments using one-way analysis of variance (ANOVA). If not, they were analyzed by the Kruskal-Wallis non-parametric test. If either of the tests showed a significant difference among the groups, the data were analyzed by the multiple comparison procedure of the Dunnett’s. Sperm motility rate, copulation index, pregnancy rate, etc. were analyzed using Fisher’s exact probability test. Litter proportions of intrauterine data and fetal malformations were compared using the Kruskal-Wallis test and the Mann-Whitney U test. The level of significance was preset at *p* < 0.05.

## Results

### Fertility and Early Embryonic Development Study

All animals survived to scheduled sacrifice. In the clinical observation, test article-related listlessness and tachypnea were observed in one male on D15 and in fifteen pregnant females on GD6 at high dose of 1.2 × 10^7^/kg, of which 4 pregnant females also had hematuria. The above clinical signs disappeared the next day. In addition, slightly thin appearance in three males at high dose and one male of vehicle control group during the administration were observed.

**FIGURE 1:**
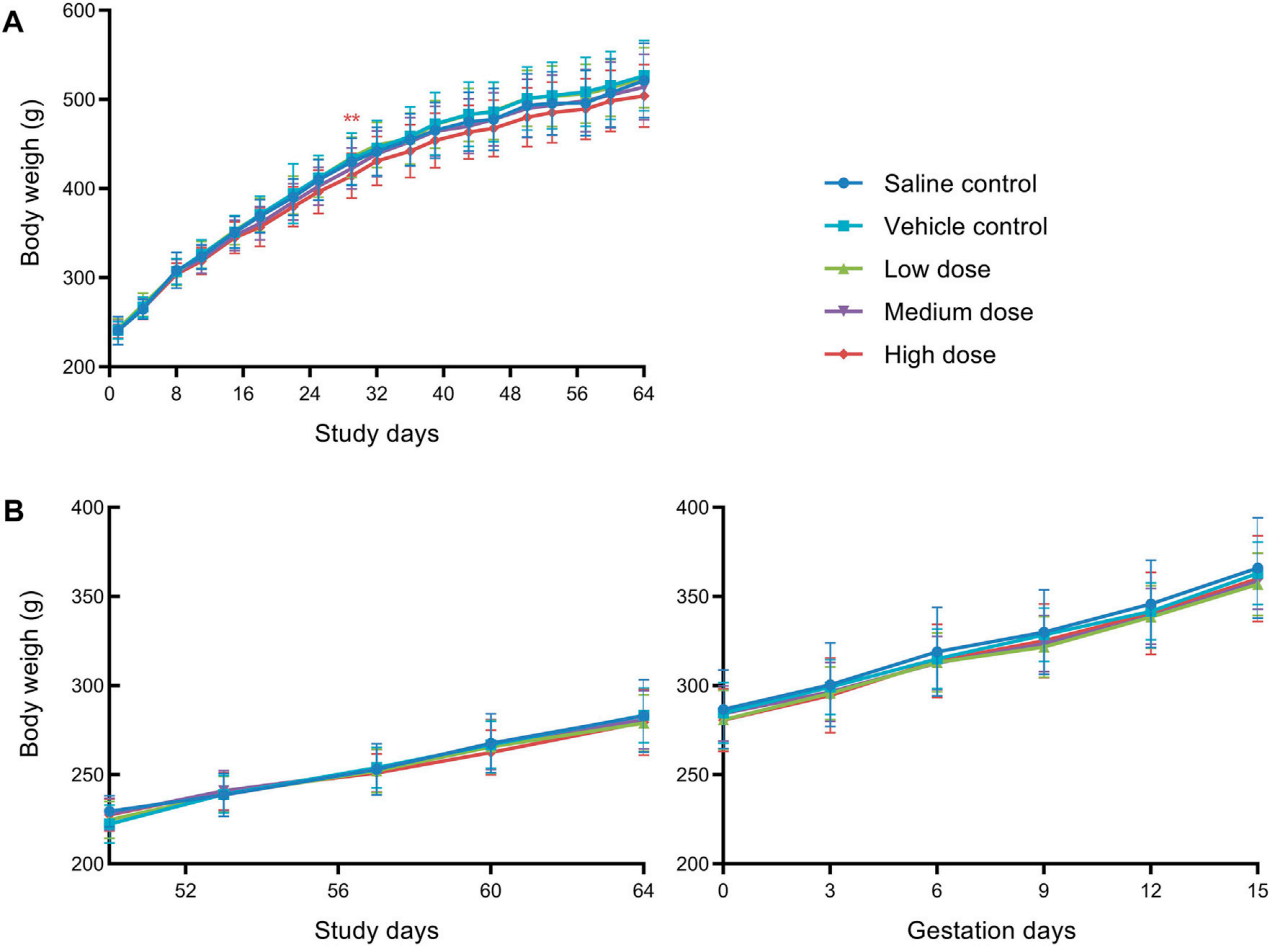
Mean body weight of male rats **(A)** and female rats **(B)** in fertility and early embryonic development study. **Significantly different from the vehicle control at *p* < 0.01.

Statistical evaluation of the body weights of males and females showed no significant difference among groups at all time points during the study, except that males in high dose group displayed significantly decreased body weights on D28 as compared to vehicle control males. As shown in Figure 1, mean body weights of males at high dose were mildly lower than those of the vehicle control males throughout the treatment period, and it is considered that hUC-MSCs at high dose of 1.2 × 10^7^/kg could reduce the body weight gain of male rats.

**FIGURE 2:**
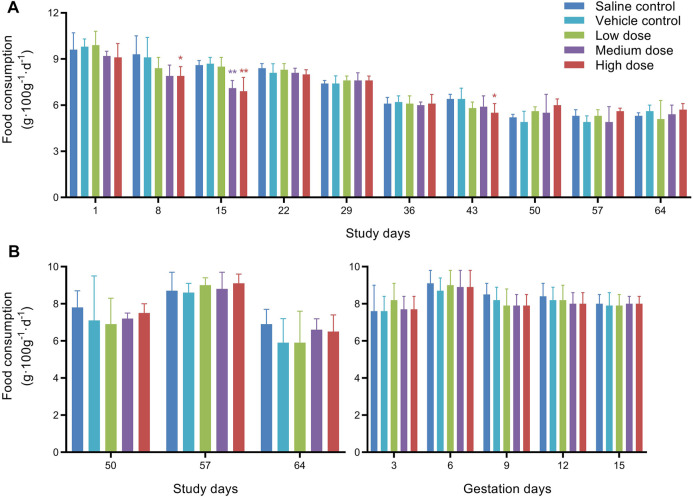
Mean food consumption of male rats **(A)** and female rats **(B)** in fertility and early embryonic development study. *Significantly different from the vehicle control at *p* < 0.05, **Significantly different from the vehicle control at *p* < 0.01.

Food consumption of the males and females is presented in Figure 2. Compared with the vehicle control group, the mean food consumption of males in the medium dose group on D15, and in the high dose group on D8, D15, and D43 were statistically significantly decreased. Considering the time of administration (D1, D8, D15, D43, D50, D57), and the mean food consumption did not change significantly during the interval of 3 weeks without dosing, the reduction of the mean food consumption in the high dose group only occurred during the administration period. Although not every high-dose administration resulted in reducing food consumption, there is still a noticeable correlation between the two. Therefore, we speculated that high doses of hUC-MSCs might reduce the food consumption of males.

Overall, there were no significant differences among groups in the measurements of weights of testes and epididymis, sperm counts, sperm motility and sperm deformation rate (Table 1). Statistically significant changes of sperm motility were observed in the low dose group compared with vehicle control group, which were not considered toxicologically meaningful since they did not exhibit a dose response relationship. Similarly, the cesarean section and fertility data of females, including corpora lutea, implantation sites, pre- and post-implantation loss, viable, dead and absorption fetuses, copulation index, and pregnancy rate, showed no test article-related changes in all dose groups (Table 2).

**TABLE 1:** Summary of male organ weights and sperm parameters from the male fertility study.

	Saline control	Vehicle control	Low dose	Medium dose	High dose
Organ weights					
Left testis (g)	1.835 ± 0.190	1.929 ± 0.167	1.906 ± 0.131	1.914 ± 0.250	1.876 ± 0.199
Right testis (g)	1.835 ± 0.191	1.923 ± 0.162	1.897 ± 0.108	1.919 ± 0.244	1.873 ± 0.197
Left epididymis (g)	0.764 ± 0.099	0.785 ± 0.095	0.762 ± 0.076	0.752 ± 0.071	0.743 ± 0.060
Right epididymis (g)	0.761 ± 0.095	0.797 ± 0.099	0.775 ± 0.082	0.770 ± 0.067	0.756 ± 0.065
Organ weights relative to body weight					
Left testis	0.353 ± 0.039	0.364 ± 0.039	0.367 ± 0.032	0.371 ± 0.055	0.372 ± 0.034
Right testis	0.352 ± 0.040	0.363 ± 0.038	0.365 ± 0.029	0.372 ± 0.055	0.372 ± 0.039
Left epididymis	0.147 ± 0.019	0.148 ± 0.019	0.147 ± 0.016	0.146 ± 0.018	0.148 ± 0.014
Right epididymis	0.146 ± 0.019	0.151 ± 0.022	0.149 ± 0.018	0.149 ± 0.015	0.150 ± 0.014
Sperm count/epididymis weight (10^7^/g)	4.951 ± 1.379	5.303 ± 1.344	5.019 ± 0.994	4.877 ± 1.084	5.043 ± 1.051
Sperm motility					
Grade Ⅰ	649 (13.0%)	718 (14.4%)	750 (15.0%)^a^	626 (12.5%)	718 (14.4%)
Grade Ⅱ	1473 (29.5%)	1445 (28.9%)	1542 (30.8%)^a^	1508 (30.2%)	1440 (28.8%)
Grade Ⅲ	1772 (35.4%)	1809 (36.2%)	1860 (37.2%)^a^	1835 (36.7%)	1824 (36.5%)
Grade Ⅳ	1106 (22.1%)	1028 (20.6%)	848 (17.0%)^a^	1031 (20.6%)	1018 (20.4%)
Sperm morphology					
Deformed sperm count	1719	1154	1079	1616	1118
Examined sperm count	25000	25000	25000	25000	25000
Deformation rate (‰)	68.76 ± 116.67	46.16 ± 12.04	43.16 ± 12.81	64.64 ± 89.46	44.72 ± 11.72

a Significantly different from the vehicle control at *p* < 0.01.

**TABLE 2:** Summary of cesarean section and fertility data from the female fertility and early embryonic development study.

	Saline control	Vehicle control	Low dose	Medium dose	High dose
Corpora lutea count	20.0 ± 3.0	19.9 ± 2.9	19.9 ± 2.4	20.2 ± 2.5	19.4 ± 1.9
Implantation sites count	16.0 ± 4.4	16.7 ± 3.0	17.2 ± 2.3	17.0 ± 1.9	16.8 ± 2.9
Preimplantation loss rate (%)	21.8 ± 15.8	16.0 ± 10.7	13.1 ± 9.4	15.3 ± 7.2	13.7 ± 11.2
Postimplantation loss rate (%)	7.0 ± 13.7	6.8 ± 10.4	8.5 ± 7.8	4.5 ± 7.0	11.5 ± 16.5
Viable fetal count	15.0 ± 4.9	15.5 ± 3.2	15.8 ± 2.8	16.3 ± 2.3	15.0 ± 4.2
Uterine fetal weight (g)	20.202 ± 6.455	21.518 ± 3.957	21.756 ± 3.045	21.721 ± 3.231	21.059 ± 5.302
Viable fetal rate (%)	93.0 ± 13.7	93.28 ± 10.4	91.5 ± 7.8	95.5 ± 7.0	88.5 ± 16.5
Dead fetal rate (%)	7.0 ± 13.7	6.8 ± 10.4	8.5 ± 7.8	4.5 ± 7.0	11.5 ± 16.5
Absorption fetal rate (%)	6.3 ± 11.9	6.5 ± 9.6	8.5 ± 7.8	4.5 ± 7.0	7.6 ± 9.0
Estrus (days)	5.0 ± 0.9	4.6 ± 0.6	4.7 ± 0.8	4.8 ± 1.5	4.7 ± 0.7
Copulation index (%)	88.0	96.0	96.0	100.0	100.0
Pregnancy rate (%)	95.5	100.0	87.5	92.0	100.0
Litter rate with dead fetuses (%)	57.1	50.0	33.3	59.1	32.0

### Embryo-Fetal Development Study

All animals survived to scheduled sacrifice with the exception of five pregnant females at high dose of 2.4 × 10^7^/kg. The deaths occurred from GD9 to GD15, all of which were acute death after administration.

**FIGURE 3:**
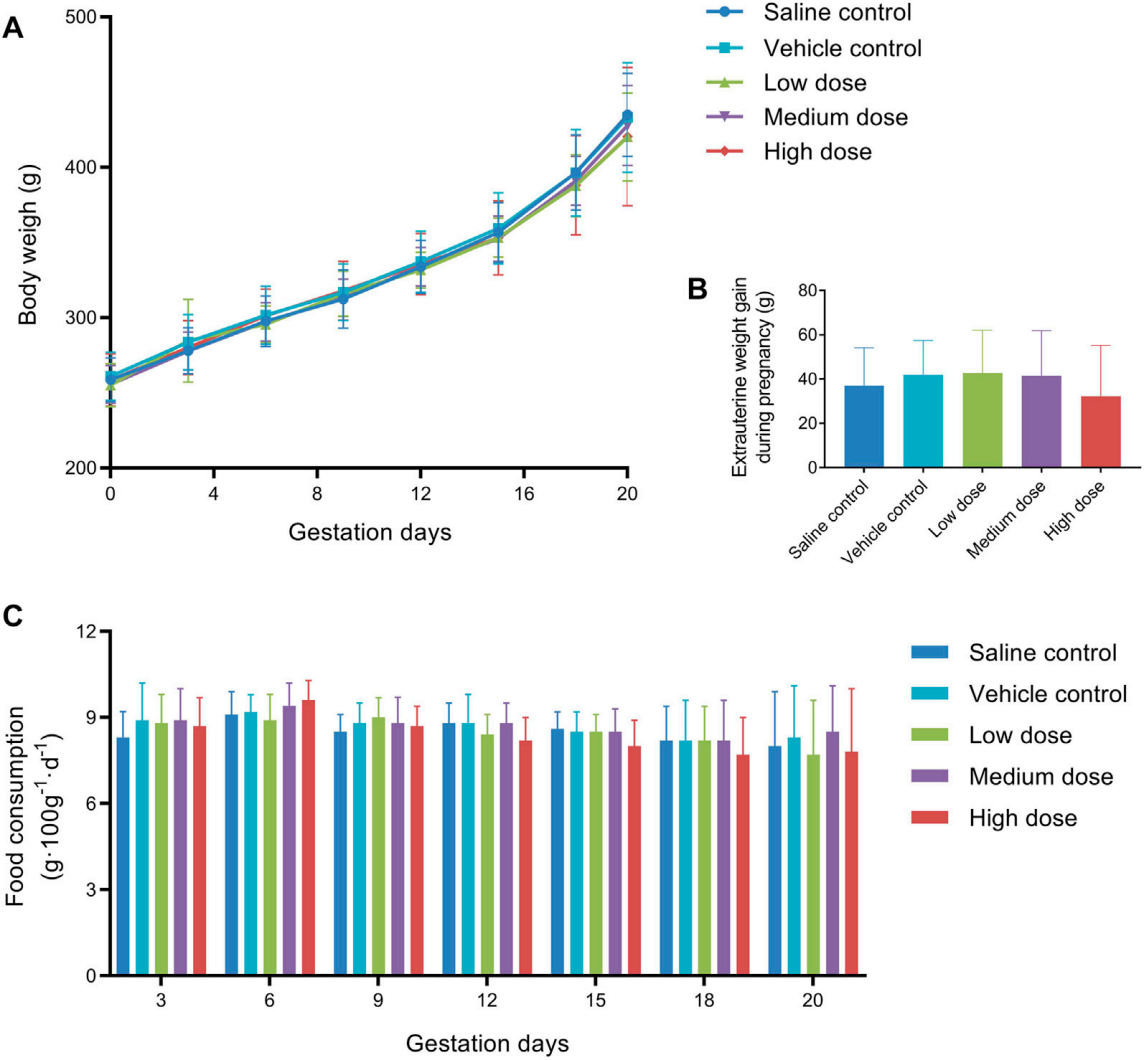
Mean body weight **(A)**, extrauterine weight gain **(B)**, and food consumption **(C)** of pregnant female rats in embryo-fetal development study.

Clinical observations of the females during the gestation periods did not show any differences in the animals’ appearance, general condition or behavior among the treatment and control groups. As shown in Figure 3A, the gestational body weights of the pregnant females showed a gradual increase during pregnancy and were not significantly different between the vehicle control group and the treated groups. Similarly, there were no differences in extrauterine weight gain (Figure 3B) or food consumption (Figure 3C).

Gestational data, including corpora lutea, implantation sites, pre- and post-implantation loss, viable, dead and absorption fetuses, is presented in Table 3. Intravenous treatment with hUC-MSCs to a dose level up 2.4 × 10^7^/kg during pregnancy did not affect these parameters. Likewise, there was no effect on the fetal sex ratio, body weight, body, and tail length (Table 3). External, visceral and skeleton examination of the fetuses showed no test article-related findings (Table 4). Visceral deformity included intraventricular hemorrhage and enlargement, renal pelvis enlargement, skeleton deformity included one extra rib, incomplete ossification of the sternum or thoracic vertebral body, residual ribs, etc., but there was no difference in the incidence of deformities in each group.

**TABLE 3:** Summary of gestational and fetal data from the pregnant female embryo-fetal development study.

	Saline control	Vehicle control	Low dose	Medium dose	High dose
Corpora lutea count	19.9 ± 2.4	18.2 ± 4.4	17.7 ± 2.8	18.4 ± 1.9	19.7 ± 3.1
Implantation sites count	17.4 ± 2.8	16.0 ± 4.2	14.0 ± 5.2	15.4 ± 4.0	16.2 ± 3.6
Preimplantation loss rate (%)	12.5 ± 9.3	11.1 ± 11.0	22.8 ± 25.6	17.3 ± 17.2	17.2 ± 14.1
Postimplantation loss rate (%)	5.8 ± 6.3	6.5 ± 9.0	3.5 ± 5.0	6.8 ± 11.2	12.8 ± 23.5
Viable fetal count	16.4 ± 2.9	14.9 ± 4.2	13.6 ± 5.2	14.2 ± 3.9	14.2 ± 5.1
Uterine fetal weight (g)	100.216 ± 18.281	89.569 ± 22.749	81.904 ± 29.345	89.090 ± 22.385	86.920 ± 30.298
Viable fetal rate (%)	94.2 ± 6.3	93.5 ± 9.0	96.5 ± 5.0	92.9 ± 11.1	87.2 ± 23.5
Dead fetal rate (%)	5.8 ± 6.3	6.5 ± 9.0	3.5 ± 5.0	7.1 ± 11.0	12.8 ± 23.5
Absorption fetal rate (%)	5.8 ± 6.3	6.2 ± 9.1	3.2 ± 4.4	6.8 ± 10.1	7.8 ± 11.8
Sex ratio (%)	1.2 ± 0.8	0.9 ± 0.6	1.3 ± 1.3	1.0 ± 0.4	0.9 ± 0.6
Placenta weight (g)	0.509 ± 0.044	0.527 ± 0.171	0.540 ± 0.126	0.510 ± 0.085	0.493 ± 0.052
Fetal weight (g)	4.097 ± 0.580	4.061 ± 0.437	4.095 ± 0.517	4.225 ± 0.747	4.125 ± 0.513
Fetal body length (mm)	36.94 ± 1.77	36.76 ± 1.20	37.42 ± 2.34	37.69 ± 2.82	37.12 ± 2.63
Fetal tail length (mm)	14.92 ± 0.77	14.82 ± 1.23	14.84 ± 0.69	15.27 ± 1.01	15.01 ± 0.72

**TABLE 4:** Effect of hUC-MSCs on fetal external, viscus and skeleton deformity.

	Saline control	Vehicle control	Low dose	Medium dose	High dose
External deformity					
Examined fetuses/litters	344/21	298/20	285/21	313/22	241/16
Deformed fetuses/litters	0/0	0/0	0/0	0/0	0/0
External deformity rate (%)	0	0	0	0	0
Visceral deformity					
Examined fetuses/litters	165/21	143/19	137/20	149/22	116/16
Deformed fetuses/litters	4/4	3/3	4/3	4/4	3/3
Viscus deformity rate (%)	2.8 ± 6.2	1.8 ± 4.3	2.8 ± 7.4	2.7 ± 6.5	2.4 ± 5.1
Skeleton deformity					
Examined fetuses/litters	179/21	155/20	14/21	164/22	125/16
Deformed fetuses/litters	0/0	1 (1)	1 (1)	0/0	0/0
Skeleton deformity rate (%)	0	0.5 ± 2.2	0.6 ± 2.7	0	0
Variable fetuses/litters	23/9	14/9	21/9	19/10	16/6
Skeleton variation rate (%)	12.5 ± 15.6	9.2 ± 12.9	12.4 ± 16.6	9.8 ± 13.5	12.4 ± 18.8

**FIGURE 4:**
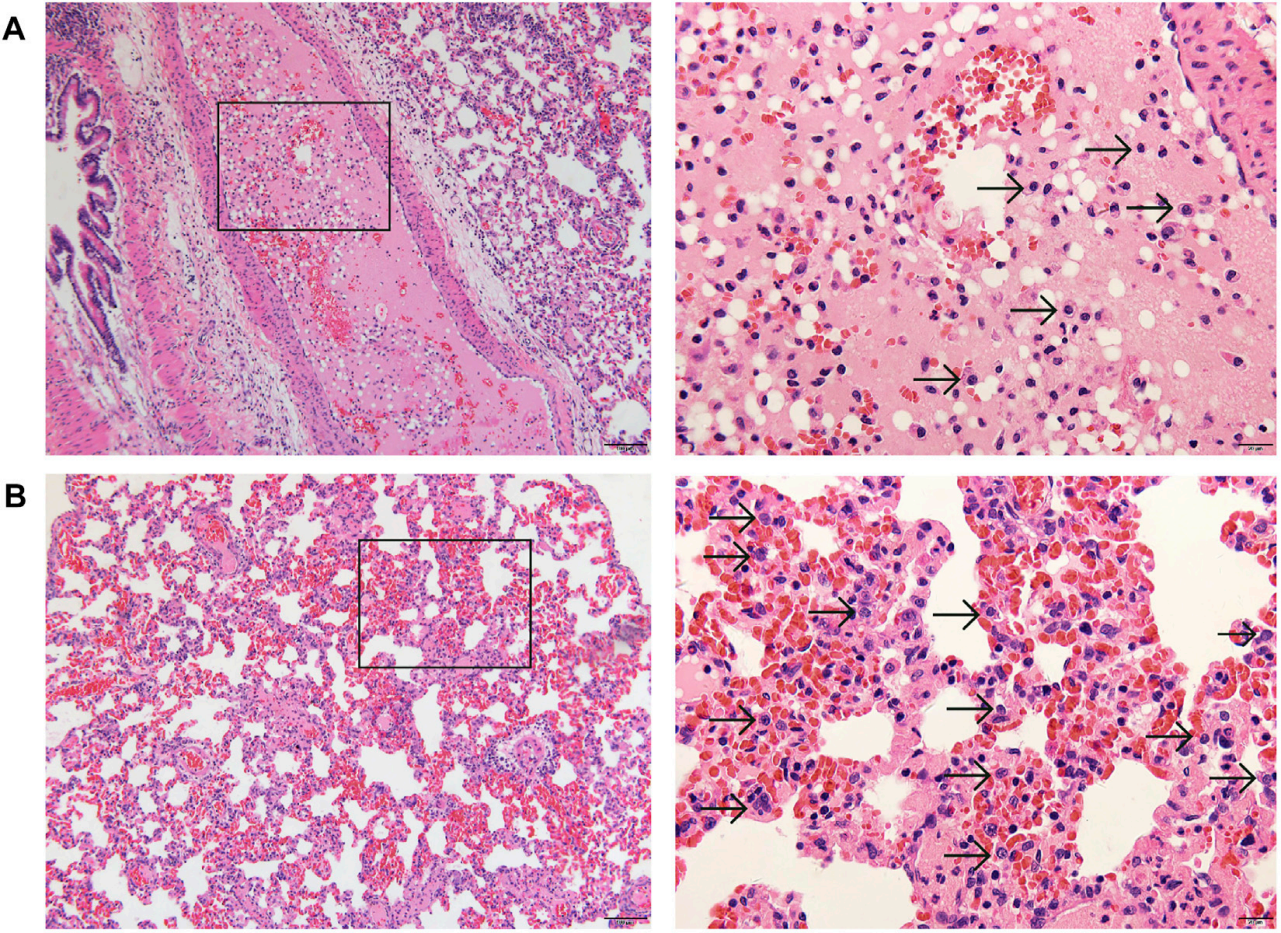
Representative histopathology of lung (left: ×100, HE; right: ×400, HE) of dead pregnant female rats at high dose of hUC-MSCs in embryo-fetal development study. A few stem cell-like cells were found in the pulmonary arterioles **(A)**, and more stem cell-like cells in the alveolar wall **(B)**. Arrows indicate the stem cell-like cells.

The necropsies were performed on five dead females, and following organs were collected and fixed: heart, aorta, liver, spleen, lung, trachea, kidneys, bladder, esophagus, stomach, duodenum, jejunum, ileum, colon, cecum, rectum, pancreas, salivary gland, eyeball, brain, hypophysis, spinal cord, ischiadic nerve, skeletal muscle, thyroid, parathyroid, adrenal gland, thymus, breast, uterus, cervix, vagina, oviduct, ovaries, sternum, femur, inguinal lymph nodes, and skin. The tissues processed by routine paraffin embedding and sections were stained with hematoxylin and eosin (HE) and subjected to histopathological examination. The histopathological findings are summarized in Table 5. Congestion of adrenal glands, liver, kidneys, and ovaries, which was considered to be caused by blood stasis after death. Thymus hemorrhage was considered as a stress response to animal death. Thyroid ultimobranchial gland residue could be a spontaneous disease. As shown in the left column of Figure 4, stem cell-like cells were found in the pulmonary arterioles (Figure 4A) and alveolar walls (Figure 4B) of dead rats under low magnification (×100), and undifferentiated stem cell-like cells with oval nuclei (indicated by the arrows) were obviously seen under high magnification (×400) in the right column of Figure 4. Stem cell-like cells were found in the alveolar walls or pulmonary arterioles of 3 dead females, suggesting that large amounts of stem cells pass through the systemic circulation into lung, causing pulmonary embolism, and then leading to the death of the animals. Although there were no obvious stem cell-like cells in the alveolar wall or pulmonary arterioles of the other two rats, the cause of death might be other local embolism in lung.

**TABLE 5:** The histopathological findings of dead pregnant female rats in embryo-fetal development study.

Organ lesion	Animal no.				
	H101	H102	H106	H119	H123
Liver congestion	+	+	+	+	+
Kidney congestion	+	+	++	++	++
Adrenal gland congestion	+	/	/	/	+
Ovarian congestion	/	/	+	/	+
Thymic hemorrhage	/	/	+	/	+
Thyroid ultimobranchial gland residue	/	/	+	/	+
Stem cell-like cells in the alveolar wall and/or pulmonary arterioles	++	/	+	+	/

+, mild; ++, moderate; /, no finding.

## Discussion

The experimental results showed that hUC-MSCs have no significant effect on the fertility of male rats, such as mating, sperm count/epididymis weight, sperm motility rate, sperm deformity rate, etc. But the body weight gain of males at high dose of 1.2 × 10^7^/kg was mildly reduced. There was no test article related effect on the fertility and early embryonic development of female rats, including body weight, food consumption, estrus, copulation index, pregnancy rate, etc., except at high dose of 1.2 × 10^7^/kg, pregnant rats suffered from transient listlessness, tachypnea and hematuria symptoms due to massive infusion of stem cells. Therefore, the no-observed-adverse-effect level (NOAEL) of fertility and early embryonic development study in rats was established at medium dose of 8.5 × 10^6^/kg. While in the embryo-fetal development study, no toxicological-relevant differences were observed in surviving pregnant female rats receiving 6.0 × 10^6^, 1.2 × 10^7^ or 2.4 × 10^7^/kg of hUC-MSCs compared with those receiving the vehicle only, including body weight, food consumption, litter, and fetus parameters, fetal external, viscus and skeleton deformity, etc., indicated that hUC-MSCs had no obvious effect on the pregnancy outcome of rats, and no teratogenic effect on fetuses. So the NOAEL of embryo-fetal development was considered as 2.4 × 10^7^/kg. The NOAEL of maternal toxicity was estimated to be 1.2 × 10^7^/kg because death due to pulmonary embolism occurred at 2.4 × 10^7^/kg.

The most common route of administration of MSCs therapy is intravascular infusion. In recent years, it has been reported in animal experiments and clinical trials that intravascular infusion of MSCs can cause a few varying degrees of pulmonary embolism and vascular embolism (Jung et al., 2013; Boltze et al., 2015; Wu et al., 2017; George et al., 2018). After MSCs were infused intravenously into the systemic circulation, most of the cells were trapped in the lung, while after arterial injection, MSCs would cause microthrombosis and blood flow obstruction (Masterson et al., 2019). When a large number of MSCs are retained in the lungs, they can cause thrombosis in local small blood vessels, but generally do not cause obvious lung tissue damage or functional impairment. Therefore, this may not be the toxic effect of MSCs, but is related to the infusion concentration, velocity and suspension uniformity of MSCs (Tian et al., 2019). Pulmonary or vascular embolism is a complex process, and its molecular mechanism is still unclear.

In the present fertility and early embryonic development study, the weight of females was lower than that of males, resulting in corresponding smaller injection of hUC-MSCs, and therefore no toxic effect of hUC-MSCs on females has been observed. However, the females gained weight rapidly due to pregnancy. When the pregnant females were injected with high doses of hUC-MSCs (1.2 × 10^7^/kg), large amounts of stem cells pass through the systemic circulation into lung, causing local pulmonary embolism, leading to listlessness, and tachypnea. Some pregnant females also occurred hematuria may be for the following reasons. The local pulmonary embolism through the pulmonary-renal artery reflex caused a decrease in renal blood flow, renal vascular ischemia, and hypoxia, resulting in acute kidney damage. Due to the increased permeability of the glomerular filtration membrane, when blood flows through the glomeruli, red blood cells could flow out of the glomerular filtration membrane and be excreted through the urinary system to form hematuria. The above symptoms are transient, and the animals can return to normal after a short rest. Probably because hUC-MSCs disappeared from the lungs within hours or migrated to other tissues such as the spleen and liver (Devine et al., 2003). Furthermore, when pregnant rats were injected with higher doses of hUC-MSCs (2.4 × 10^7^/kg) in the embryo-fetal development study, local pulmonary embolism could lead to death, which was similar to the previous acute study of hUC-MSCs in mice that death occurred when the injection dose reached 1.4 × 10^8^/kg (Hai et al., 2016). The pulmonary embolism toxicity in these two studies all occurred in the high-dose group of animals (1.2 × 10^7^–2.4 × 10^7^/kg), and the clinically applied dose is generally 1 × 10^6^–2 × 10^6^/kg, so the clinical risk of hUC-MSCs transvascular infusion is relatively low.

The male rats showed mildly reduced body weight gain at high dose of hUC-MSCs (1.2 × 10^7^/kg) in fertility and early embryonic development study, but these did not occur in the females. One reason may be that the females weighed much less than the males, resulting in a correspondingly less amount of injected hUC-MSCs. Another reason may be that the administration period of the males was longer than that of the females. Therefore, the development of males was more affected by high-dose hUC-MSCs than the females. Additionally, the food consumption of males was reduced when high-dose hUC-MSCs were administered (D8, D15, and D43). Inadequate nutritional intake may be a contributing factor of the body weight loss, so it was presumed that the mild reduction in body weight of males at high dose was partly related to the decline in food consumption.

A limitation of the two studies was the absence of the accompanied toxicokinetics (TK) evaluation, which resulted in a lack of TK data on parental systemic exposure and fetuses, leading to uncertainty about the relationship between systematic exposure achieved in rats and dose level, exposure time and toxicological results, and whether hUC-MSCs had crossed the placental barrier. These data can be used to interpret toxicology findings and the relevance to clinical safety issues. In our study, no toxicity of hUC-MSCs was observed in the fetuses, probably because the placental barrier blocks the invasion of hUC-MSCs and thus protects the fetuses from hUC-MSCs. Previously, we have carried out hUC-MSCs repeated dose toxicity study in cynomolgus monkeys with accompanied TK. hUC-MSCs were administered intravenously every 5 days at two dose levels (2 × 10^6^/kg and 1 × 10^7^/kg). After 9 consecutive administrations, although hUC-MSCs accumulated in the blood of monkeys, its did not cause obvious toxicity (unpublished data). Besides, the numbers of hUC-MSCs in the testis or ovaries were not determined in our experiments, which is also a deficient of our experimental design. As reported, intravenously injected hUC-MSCs have been evidenced to be able to migrate to the ovary tissue in the premature ovarian failure rats (Zhu et al., 2015; Deng et al., 2021).

Currently, hUC-MSCs are used in clinical trials to treat a variety of diseases, such as liver diseases, immune system diseases, diabetes, severe COVID-19, etc. In the future, hUC-MSCs may be used in pregnant women and women with childbearing potential, especially when it is used to treat people with systemic lupus erythematosus or lupus nephritis which is more prevalent in women of childbearing age (En-Nasri et al., 2014; Fischer-Betz and Specker, 2017). Therefore, it is important to determine the reproductive and developmental effects of hUC-MSCs therapy related to human risk. Since data available on the reproductive and developmental toxicity of hUC-MSCs is scarce, our present studies could provide preliminary information to fill this gap.

In summary, no hUC-MSCs-related toxicities was observed on the fertility of male and female rats, and no teratogenic effect on fetuses. hUC-MSCs at 1.2 × 10^7^/kg caused a mildly decrease in body weight gain of male rats, transient listlessness, tachypnea and hematuria symptoms in pregnant female rats. Death was observed in part of the pregnant females at a dose of 2.4 × 10^7^/kg, which could be due to pulmonary embolism. The two reproductive and developmental toxicity studies in rats demonstrated that hUC-MSCs has a low potential to produce effects on reproduction or development. These results will be used as initial information about the toxicological properties of hUC-MSCs in reproduction and development. Further toxicity studies, such as prenatal/postnatal toxicity study, should be undertaken to understand better the effects of hUC-MSCs on human reproduction and development.

## Conclusion

Based on the results of the studies, no hUC-MSCs-related toxicity was observed on the fertility of male and female rats, and no teratogenic effect on fetuses. The no-observed-adverse-effect levels (NOAELs) are 8.5 × 10^6^/kg for fertility and early embryonic development, 1.2 × 10^7^/kg for maternal toxicity and 2.4 × 10^7^/kg for embryo-fetal development in rats intravenous injected with hUC-MSCs, which are equivalent to 8.5-fold, 12-fold, and 24-fold respectively of its clinical dosage in humans. hUC-MSCs at 1.2 × 10^7^/kg caused a mildly decrease in body weight gain of male rats, transient listlessness, tachypnea and hematuria symptoms in pregnant female rats. Death was observed in part of the pregnant females at a dose of 2.4 × 10^7^/kg, which could be due to pulmonary embolism.

## Data Availability

The original contributions presented in the study are included in the article/Supplementary Material, further inquiries can be directed to the corresponding authors.
